# Advances and Prospects of Nanomaterials for Solid-State Hydrogen Storage

**DOI:** 10.3390/nano14121036

**Published:** 2024-06-16

**Authors:** Yaohui Xu, Yuting Li, Liangjuan Gao, Yitao Liu, Zhao Ding

**Affiliations:** 1 Laboratory for Functional Materials, School of New Energy Materials and Chemistry, Leshan Normal University, Leshan 614000, China; 2 Leshan West Silicon Materials Photovoltaic New Energy Industry Technology Research Institute, Leshan 614000, China; 3 College of Materials Science and Engineering, National Engineering Research Center for Magnesium Alloys, National Innovation Center for Industry-Education Integration of Energy Storage Technology, Chongqing University, Chongqing 400044, China; 4 College of Materials Science and Engineering, Sichuan University, Chengdu 610065, China; 5 Department of Electrical and Computer Engineering, Illinois Institute of Technology, Chicago, IL 60616, USA

**Keywords:** hydrogen storage, nanomaterials, size effect, surface modification, nanocomposites, nanocatalysts

## Abstract

Hydrogen energy, known for its high energy density, environmental friendliness, and renewability, stands out as a promising alternative to fossil fuels. However, its broader application is limited by the challenge of efficient and safe storage. In this context, solid-state hydrogen storage using nanomaterials has emerged as a viable solution to the drawbacks of traditional storage methods. This comprehensive review delves into the recent advancements in nanomaterials for solid-state hydrogen storage, elucidating the fundamental principles and mechanisms, highlighting significant material systems, and exploring the strategies of surface and interface engineering alongside catalytic enhancement. We also address the primary challenges and provide future perspectives on the development of nanomaterial-based hydrogen storage technologies. Key discussions include the role of nanomaterial size effects, surface modifications, nanocomposites, and nanocatalysts in optimizing storage performance.

## 1. Introduction

Hydrogen, as a clean and sustainable energy carrier, has attracted tremendous attention in recent years due to the increasing concerns over energy shortages and environmental issues caused by the excessive consumption of fossil fuels [1,2]. With the highest gravimetric energy density (142 MJ/kg) among all chemical fuels, hydrogen is considered a promising alternative to traditional energy sources for various applications, especially in the transportation sector [3]. However, the widespread use of hydrogen energy is severely hindered by the lack of efficient and safe hydrogen storage technologies [4].

In the development of hydrogen storage technologies, various storage methods exhibit distinct characteristics, with differences in storage density, operating conditions, safety, cost, and applicable scenarios. High-pressure hydrogen storage offers the advantage of rapid release and flexible application but comes with high equipment costs and safety risks, making it suitable for mobile applications such as fuel cell vehicles. Liquid hydrogen storage boasts high storage density and is suitable for large-scale transport, but it requires extremely low temperatures, high energy consumption, and elevated costs, making it more applicable for industrial-scale hydrogen transportation. Liquid organic hydrogen carriers (LOHCs) are convenient to operate at ambient temperature and pressure, are easy to store and transport, and offer good safety, suitable for high-flexibility, distributed applications. However, they have lower hydrogen storage density and complex dehydrogenation processes [5]. Solid-state hydrogen storage, with its high storage density and safety, involves storing hydrogen within or on the surface of materials through physical adsorption, chemical adsorption, or chemical reactions. It is suitable for applications requiring long-term stable hydrogen storage in limited space [6,7].

Compared to other storage methods, solid-state hydrogen storage has significant advantages in terms of high density and safety, making it a promising emerging technology in the hydrogen storage and transportation sector. To expand its applicability and accelerate commercialization, challenges such as the need for high temperatures or catalysts for hydrogen release and the high cost of materials need to be addressed. Therefore, developing advanced hydrogen storage materials and technologies is crucial for the transition to a hydrogen-based energy economy. Nanomaterials have shown great promise for solid-state hydrogen storage due to their unique size-dependent properties and large surface-to-volume ratios [8]. The reduced size and dimensionality of nanomaterials can lead to significantly enhanced hydrogen storage capacities and kinetics compared to their bulk counterparts [3,9]. Moreover, the high specific surface area and abundant surface sites of nanomaterials facilitate the adsorption and dissociation of hydrogen molecules, enabling fast hydrogen uptake and release [10]. The versatile surface chemistry of nanomaterials also allows for the tuning of their hydrogen storage properties through surface modification and functionalization [11,12]. With these advantages, nanomaterials have emerged as a frontier in the research and development of advanced hydrogen storage technologies.

In recent years, significant progress has been made in the development of nanomaterials for solid-state hydrogen storage, a crucial area for advancing clean energy technologies. This review provides a comprehensive overview of these advancements, beginning with an introduction to the fundamental principles and mechanisms of hydrogen storage in nanomaterials. Emphasizing the effects of size, surface, and interface on hydrogen storage performance, we lay the groundwork for understanding how these factors influence efficiency and capacity. We then explore various nanomaterial systems used for hydrogen storage, including light-weight metal nanoparticles, porous nanomaterials, and other emerging platforms, highlighting their unique properties and potential applications. Strategies for enhancing hydrogen storage properties through surface and interface engineering, such as surface modification, nanocomposite construction, and defect engineering, are discussed in detail, showcasing their impact on storage capabilities. The role of nanocatalysts in promoting hydrogen storage kinetics and their catalytic mechanisms is also reviewed, emphasizing the importance of these processes in developing faster and more efficient storage systems. Finally, we summarize key progress and remaining challenges in the field of nanomaterial-based hydrogen storage and provide an outlook on future developments, offering insights into potential directions and innovations that could drive further advancements in this technology. By consolidating recent research and developments, this review aims to serve as a valuable resource for researchers and engineers working towards the realization of efficient and scalable hydrogen storage solutions.

## 2. Principles and Mechanisms of Hydrogen Storage in Nanomaterials

**Figure 1:**
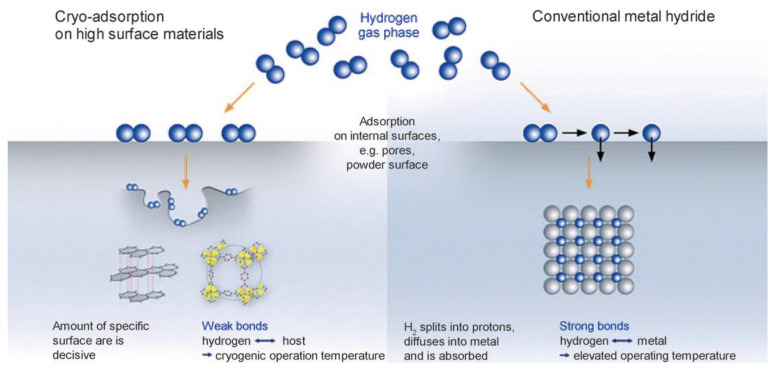
Schematic diagrams illustrating the principles of physical hydrogen storage and chemical hydrogen storage [2].

Solid-state hydrogen storage technology achieves hydrogen energy storage by storing hydrogen in solid materials, relying on physical and chemical adsorption processes. Specifically, this technology depends on specific solid materials, such as porous adsorbents and metal hydrides, to capture and release hydrogen. The primary adsorption mechanisms are illustrated in Figure 1 [2]. Physical adsorbents, such as metal-organic frameworks (MOFs) and activated carbon, utilize their high surface area and microporous structures to adsorb hydrogen molecules on their surface via van der Waals forces. These materials typically exhibit high hydrogen storage capacity at low temperatures, and their storage performance can be further enhanced by modifying the structure and surface properties of the materials. Metal hydrides, on the other hand, are materials that chemically react with hydrogen to form stable compounds. Typical metal hydrides include TiFe-based alloys and LaNi_5_, which form hydrides upon hydrogen absorption and release hydrogen upon heating or pressure reduction. During hydrogen absorption, metal hydrides absorb heat, while they release heat during hydrogen desorption, making precise control of temperature and pressure conditions crucial for efficient hydrogen adsorption and release.

### 2.1. Size and Surface Effects on Hydrogen Storage Properties

The hydrogen storage properties of nanomaterials are strongly dependent on their size and surface characteristics. As the size of a material decreases to the nanoscale, its surface-to-volume ratio increases dramatically, leading to a higher fraction of surface atoms or molecules [13]. These surface species often possess unsaturated coordination environments and exhibit distinct electronic, structural, and chemical properties compared to their bulk counterparts [14]. In the context of hydrogen storage, the unique surface properties of nanomaterials can significantly influence their interaction with hydrogen molecules and the corresponding storage performance.

One of the most prominent size effects in nanomaterial-based hydrogen storage is the enhanced hydrogen adsorption capacity. Theoretical and experimental studies have shown that the hydrogen uptake of nanomaterials increases with decreasing particle size [15,16]. Using appropriate nanocatalysts for hydrogen adsorption and/or reducing the particle size of MgH_2_ to the nanoscale has proven to be an effective strategy [17]. The enhanced hydrogen storage capacity of nanomaterials can be attributed to several factors. First, the large surface area of nanomaterials provides more adsorption sites for hydrogen molecules, leading to increased hydrogen uptake [18]. Second, the surface energy of nanomaterials increases with decreasing particle size, which can lower the activation barrier for hydrogen adsorption and facilitate hydrogen diffusion [19]. Third, the nanoscale size can induce structural changes, such as increased lattice distortions and defects, which may create additional hydrogen trapping sites and enhance the hydrogen storage capacity [20]. The size effect on hydrogen storage properties can be attributed to several factors. First, as the particle size decreases, the surface-to-volume ratio increases dramatically, leading to a higher fraction of surface atoms with unsaturated coordination environments. These surface atoms can act as additional adsorption sites for hydrogen molecules, thereby enhancing the storage capacity. Second, the increased surface energy and curvature of nanoparticles can modulate the electronic structure and reactivity of the material, facilitating hydrogen adsorption and dissociation [21]. Third, the nanoscale confinement effect can alter the thermodynamics and kinetics of hydrogen sorption reactions, enabling faster kinetics and improved reversibility [22].

In addition to the size effect, the surface chemistry of nanomaterials also plays a crucial role in their hydrogen storage properties. The surface of nanomaterials can be modified with various functional groups, which can alter their interaction with hydrogen molecules and influence the hydrogen adsorption/desorption behavior. The metal nanoparticles act as catalytic sites for the dissociation of hydrogen molecules, which then spill over onto the CNT surface and diffuse into the bulk, leading to increased hydrogen uptake. Similarly, the modification of metal-organic frameworks (MOFs) with functional groups, such as amine and hydroxyl groups, has been shown to improve their hydrogen storage capacity by providing additional binding sites for hydrogen molecules [23].

The surface composition and structure of nanomaterials can also affect their hydrogen storage performance. For instance, the presence of surface oxides or hydroxides on metal nanoparticles can hinder the hydrogen adsorption process by forming a barrier layer [24]. On the other hand, the introduction of surface defects, such as vacancies and edges, can create active sites for hydrogen adsorption and enhance the hydrogen storage capacity [25,26]. Therefore, controlling the surface composition and creating desired surface structures are important strategies for optimizing the hydrogen storage properties of nanomaterials.

### 2.2. Key Performance Indicators and Characterization Techniques

To evaluate the hydrogen storage performance of nanomaterials, several key performance indicators need to be considered, including gravimetric and volumetric storage capacities, thermodynamics, kinetics, and cyclability [27].

Gravimetric storage capacity, also known as specific capacity, refers to the amount of hydrogen stored per unit mass of the material (wt.%). It is one of the most important parameters for hydrogen storage materials, especially for mobile applications where the weight of the storage system is a critical factor. The U.S. Department of Energy (DOE) has set a target of 7.5 wt.% for the gravimetric capacity of onboard hydrogen storage systems by 2025. Although many nanomaterials have shown improved gravimetric capacities compared to their bulk counterparts, achieving this target remains a significant challenge.

Volumetric storage capacity, on the other hand, represents the amount of hydrogen stored per unit volume of the material (kg/m^3^). It is another crucial parameter for practical hydrogen storage applications, particularly for stationary and large-scale storage systems. The DOE target for the volumetric capacity of onboard hydrogen storage systems is 70 g/L by 2025 [28]. Nanomaterials with high porosity and surface area, such as MOFs and porous carbons, have shown promising volumetric storage capacities due to their ability to adsorb hydrogen molecules in their pores and cavities [23].

Thermodynamics and kinetics are also important factors in evaluating the hydrogen storage performance of nanomaterials. The thermodynamics of hydrogen storage determines the temperature and pressure conditions required for hydrogen adsorption and desorption [29]. Ideally, hydrogen storage materials should have a moderate binding energy with hydrogen molecules, allowing for fast and reversible hydrogen uptake and release under mild conditions. However, many nanomaterials, especially those based on chemisorption, suffer from high desorption temperatures and slow kinetics due to the strong binding of hydrogen [28]. Therefore, tuning the thermodynamics and kinetics of hydrogen storage in nanomaterials through composition and structural engineering is an active area of research.

Cyclability, which refers to the ability of a material to maintain its hydrogen storage capacity over multiple adsorption-desorption cycles, is another key performance indicator for practical applications [30]. Nanomaterials often suffer from capacity degradation during cycling due to structural changes, agglomeration, or chemical reactions with impurities [31]. Improving the cycling stability of nanomaterials through surface protection, nanostructure optimization, and impurity control is crucial for their long-term use in hydrogen storage systems.

To characterize the hydrogen storage properties of nanomaterials and understand the underlying mechanisms, various experimental techniques have been employed, including volumetric/gravimetric methods, spectroscopic techniques, and microscopic characterizations [13]. Volumetric and gravimetric methods, such as Sieverts apparatus and thermogravimetric analysis (TGA), are widely used to measure the hydrogen storage capacity and sorption kinetics of nanomaterials under different temperature and pressure conditions. Spectroscopic techniques, such as Fourier-transform infrared (FTIR) spectroscopy, Raman spectroscopy, and X-ray photoelectron spectroscopy (XPS), provide valuable information on the chemical bonding, surface composition, and oxidation states of nanomaterials during hydrogen storage. Microscopic characterizations, including scanning electron microscopy (SEM), transmission electron microscopy (TEM), and atomic force microscopy (AFM), offer direct visualization of the morphology, structure, and surface properties of nanomaterials at the nanoscale. Other techniques, such as X-ray diffraction (XRD), small-angle X-ray scattering (SAXS), and neutron scattering, are also used to probe the crystalline structure, pore size distribution, and hydrogen binding sites in nanomaterials.

In summary, understanding the fundamental principles and mechanisms of hydrogen storage in nanomaterials is crucial for the design and development of advanced hydrogen storage systems. The size and surface effects, along with the key performance indicators and characterization techniques, provide important guidelines for the rational engineering of nanomaterials with enhanced hydrogen storage properties.

According to the targets set by the U.S. Department of Energy (DOE), the gravimetric and volumetric densities of onboard hydrogen storage systems should reach 7.5 wt.% and 70 g/L, respectively, by 2025. Currently, the hydrogen storage performance of most nanomaterials still falls short of these targets. For instance, MOF-177 exhibits a gravimetric density of 7.5 wt.% at 77 K and 70 bar, but its volumetric density is only 32 g/L; the magnesium-based material MgH_2_ has a high theoretical gravimetric density of 7.6 wt.%, but due to its high thermodynamic stability, it is difficult to achieve reversible hydrogen storage at ambient conditions. Therefore, further optimization of the composition, structure, and interfaces of nanomaterials to enhance their hydrogen storage capacity and kinetic performance remains a significant challenge.

## 3. Typical Nanomaterial Systems for Hydrogen Storage

### 3.1. Lightweight Metal Nanoparticles and Their Composites

Lightweight metals, such as magnesium (Mg), aluminum (Al), and lithium (Li), have been extensively studied as potential hydrogen storage materials due to their high theoretical storage capacities and low cost [32,33]. However, their practical applications are hindered by slow kinetics, high desorption temperatures, and poor reversibility. Nanostructuring of these metals has emerged as a promising approach to overcome these limitations and enhance their hydrogen storage performance [34,35]. The performance characteristics of all kinds of light storage materials are shown in Table 1.

Magnesium-based nanomaterials have received particular attention due to their high theoretical storage capacity (7.6 wt.% for MgH_2_) and abundance. Jongh et al. [36] synthesized Mg nanocrystals with particle sizes ranging from 2 to 5 nm within carbon nanopores using a melt impregnation method, improving the hydrogen absorption and desorption kinetics. The enhanced performance was attributed to the increased surface area, shortened diffusion paths, and destabilization of the hydride phase at the nanoscale. However, the stability of Mg nanoparticles remains a challenge due to their high reactivity and tendency to form surface oxides [37]. To further improve the hydrogen storage performance of Mg-based nanomaterials, various strategies have been explored, such as alloying with transition metals, nanoconfinement in porous scaffolds [38], and surface modification with catalytic additives. For instance, Paskevicius et al. [39] successfully synthesized 2 nm MgH_2_ in carbon aerogels using a wet impregnation method. This significantly reduced the initial hydrogen desorption temperature to 220 °C, with complete hydrogen release achieved upon heating to 300 °C. Zhang et al. [40] synthesized MgH_2_@CSC composites via a solid-phase chemical reaction. These composites had an initial hydrogen desorption temperature of 245 °C and could release 5.4 wt.% hydrogen within 10 min at 325 °C. At 250 °C, the desorbed composites released 5.0 wt.% hydrogen within 5 min. The carbon matrix acted as a physical barrier to prevent the agglomeration and oxidation of Mg nanoparticles during hydrogen cycling. Similarly, the formation of Mg-based nanocomposites with transition metals, such as Ni, Fe, and Ti, has been shown to enhance the hydrogen storage kinetics and reduce the desorption temperature through catalytic effects and destabilization of the hydride phase [41].

TiFe-based hydrogen storage alloys have garnered significant attention in solid-state hydrogen storage technology due to their excellent storage performance and relatively low cost. These alloys offer high hydrogen storage capacity and good cycling stability, capable of efficiently adsorbing and releasing hydrogen at ambient temperature and pressure, significantly enhancing the safety and reliability of hydrogen storage systems [42]. The hydrogen adsorption/desorption process in TiFe-based alloys exhibits rapid kinetics, contributing to improved hydrogen utilization efficiency. However, these alloys face challenges in practical applications, such as requiring activation before initial use, which increases operational complexity [43]. Additionally, TiFe-based alloys are sensitive to impurities, particularly oxygen and water vapor, which can degrade their storage performance. Nonetheless, optimizing alloy composition and refining manufacturing processes can substantially improve their impurity resistance and cycling life. Overall, TiFe-based hydrogen storage alloys hold broad application prospects in solid-state hydrogen storage technology, but further research and optimization are needed to overcome existing technical challenges and achieve industrial-scale adoption.

Aluminum-based nanomaterials have also been investigated for hydrogen storage applications. Although the theoretical storage capacity of Al (10.1 wt.% for AlH_3_) is higher than that of Mg, its practical use is limited by the high stability of AlH_3_ and the difficulty in its regeneration [44]. Nanostructuring of Al has been shown to improve its hydrogen storage properties by destabilizing the hydride phase and facilitating the formation of metastable alane (AlH_3_) phases [44,45]. The nanoparticles also exhibited improved reversibility and faster kinetics, attributed to the increased surface area and shorter diffusion paths.

Lithium-based nanomaterials, particularly Li-N-H systems, have attracted attention due to their high theoretical storage capacity (11.5 wt.% for LiNH_2_) and low operating temperatures [46,47]. However, the slow kinetics and poor reversibility of Li-N-H systems remain significant challenges [48]. Nanostructuring of Li-N-H materials has been explored as a means to enhance their hydrogen storage performance. Osborn et al. [47] discovered that ball milling LiNH_2_ + LiH composites at low temperatures significantly increased the hydrogen desorption kinetics. Nuclear magnetic resonance analysis showed that the internal defects generated during low-temperature ball milling were retained, which were the main reasons for the accelerated kinetics of the composites.

Despite the progress made in lightweight metal nanomaterials for hydrogen storage, several challenges still need to be addressed for their practical applications. These include further improvement in the storage capacity and kinetics, as well as the development of efficient and scalable synthesis methods for high-quality nanomaterials. Moreover, the long-term stability and cyclability of these nanomaterials under realistic operating conditions need to be carefully evaluated and optimized.

### 3.2. Porous Nanomaterials (Carbon-Based Materials, MOFs/COFs, BNHs)

Porous nanomaterials, such as carbon-based materials, metal-organic frameworks (MOFs), covalent organic frameworks (COFs), and boron nitride-based materials, have been widely investigated for hydrogen storage applications due to their high surface area, tunable porosity, and versatile chemical properties [49,50]. Table 2 summarizes the main performance characteristics of various porous storage materials.

Carbon-based nanomaterials, including activated carbons, carbon nanotubes (CNTs), and graphene, have been extensively studied for hydrogen storage owing to their light weight, high surface area, and good chemical stability [51]. Activated carbons, which are typically prepared by the pyrolysis and activation of carbonaceous precursors, possess a highly developed porous structure with a large surface area (up to 3000 m^2^/g) and a wide range of pore sizes [52]. The high surface area and microporosity of activated carbons make them promising candidates for hydrogen storage through physical adsorption. However, the hydrogen storage capacity of activated carbons is relatively low (typically < 2 wt.% at room temperature and 100 bar) due to the weak interaction between hydrogen molecules and the carbon surface [53].

To enhance the hydrogen storage capacity of carbon-based materials, various strategies have been explored, such as surface modification, doping with heteroatoms, and the creation of nanostructures with optimized pore sizes. Zhou et al. [54] found that adsorbed hydrogen formed a monolayer on the carbon surface; thus, the storage capacity depended solely on the specific surface area of the carbon. If the van der Waals forces between hydrogen and the solid surface remained, this rule would also apply to other organic/inorganic materials. Due to the small surface area of carbon nanotubes, they were not suitable as hydrogen carriers, but superactivated carbon showed great potential, with a storage capacity exceeding 10 wt.% at 77 K and 6 MPa. 

Metal-organic frameworks (MOFs) are a class of crystalline porous materials composed of metal ions/clusters coordinated with organic linkers [55]. The high surface area (up to 7000 m^2^/g), tunable porosity, and diverse chemical functionalities of MOFs make them attractive candidates for hydrogen storage [56]. The hydrogen storage capacity of MOFs is influenced by several factors, including the surface area, pore size, and the presence of open metal sites and functional groups [57]. Li et al. [58] synthesized MOF-5 with different morphologies using various methods. The specific surface area and pore volume of MOF-5 were positively correlated with its hydrogen storage capacity. The study showed that under conditions of 1.74 MPa and 77 K, the hydrogen storage capacities of MOF-5 samples prepared by slow diffusion, direct mixing, and solvothermal methods were 2.63 wt.%, 3.20 wt.%, and 3.60 wt.%, respectively.

Strategies for improving the hydrogen storage capacity of MOFs include the incorporation of open metal sites, the functionalization of organic linkers, and the creation of hierarchical pore structures [59]. For instance, the introduction of unsaturated metal centers, such as Cu(II) and Ni(II), into MOFs has been shown to enhance their hydrogen adsorption capacity and binding energy due to the strong interaction between the metal sites and hydrogen molecules. The functionalization of organic linkers with polar groups, such as amine and hydroxyl groups, has also been explored to increase the hydrogen binding energy and storage capacity of MOFs. Moreover, the development of MOFs with hierarchical pore structures, consisting of both micropores and mesopores, has been demonstrated to facilitate the diffusion and accessibility of hydrogen molecules within the framework.

Covalent organic frameworks (COFs) are another class of porous materials that have gained increasing attention for hydrogen storage applications [60]. COFs are constructed from lightweight elements (C, H, N, O, B) linked by strong covalent bonds, forming crystalline and porous structures [61]. Compared to MOFs, COFs possess higher chemical and thermal stability, as well as a lower density, which are advantageous for hydrogen storage [62]. However, the hydrogen storage capacity of COFs is generally lower than that of MOFs due to their smaller surface area and pore volume [63].

To enhance the hydrogen storage performance of COFs, various strategies have been investigated, such as the design of COFs with high surface area and appropriate pore size, the incorporation of heteroatoms, and the creation of interpenetrated structures [60]. Klontzas et al. [64] conducted multi-scale structural optimization of the ultra-low-density COF-102 network. Using grand canonical Monte Carlo simulations, they verified the storage capacity of the optimized structures. One novel COF exhibited a gravimetric uptake exceeding 25 wt.% at 77 K and achieved the Department of Energy’s target of 6 wt.% at room temperature. The high capacity was attributed to the large surface area and the uniform microporous structure of the COF, which allowed for efficient adsorption of hydrogen molecules. The incorporation of heteroatoms, such as nitrogen and sulfur, into COFs has also been shown to improve their hydrogen binding energy and storage capacity by creating additional adsorption sites and increasing the polarity of the framework.

Boron nitride-based materials, particularly boron nitride nanotubes (BNNTs) and hexagonal boron nitride (h-BN), have also been explored for hydrogen storage applications [65]. BNNTs and h-BN possess similar structures to carbon nanotubes and graphene, respectively, but with alternating boron and nitrogen atoms instead of carbon [66]. The polar nature of the B-N bond and the presence of surface defects in BNNTs and h-BN have been shown to enhance their hydrogen adsorption capacity and binding energy compared to their carbon counterparts [65,66]. The high capacity was attributed to the presence of defects and the polarized surface of the BNNTs, which facilitated the adsorption and dissociation of hydrogen molecules.

Despite the promising hydrogen storage properties of porous nanomaterials, several challenges still need to be addressed for their practical applications. These include the development of cost-effective and scalable synthesis methods, the improvement of the volumetric storage capacity, and the enhancement of the hydrogen adsorption/desorption kinetics and cyclability. Moreover, the stability and safety of these materials under realistic operating conditions, such as high pressure and temperature, need to be carefully evaluated and optimized.

### 3.3. Other Emerging Nanomaterial Platforms (MXenes, LDHs)

In addition to the widely studied nanomaterial systems discussed above, several emerging nanomaterial platforms have shown promising potential for hydrogen storage applications, such as MXenes and layered double hydroxides (LDHs).

MXenes are a class of two-dimensional (2D) transition metal carbides, nitrides, or carbonitrides with a general formula of M_n_ + 1X_n_T_x_, where M is an early transition metal (e.g., Ti, V, Cr), X is carbon and/or nitrogen, and T_x_ represents the surface terminations (e.g., -OH, -O, -F) [67]. MXenes are typically synthesized by the selective etching of the A layer from their MAX phase precursors, where A is an element from group 13 or 14 (e.g., Al, Si) [68]. The unique combination of metallic conductivity, hydrophilicity, and high surface area of MXenes makes them attractive candidates for hydrogen storage [69]. Table 3 summarizes the performance characteristics of vanadium-based MXene materials.

Several studies have reported the hydrogen storage properties of MXenes, particularly Ti_3_C_2_T_x_, which is the most widely investigated MXene [70,71]. Ghotia et al. [72] synthesized 2D Ti_3_C_2_T_x_ MXenes via an etching method. The hydrogen storage capacity of multilayer Ti_3_C_2_T_x_ MXenes was measured to be ~10.47 wt.% at 25 bar and 77 K, revealing an extraordinary hydrogen uptake in the multilayer MXene samples.

To further enhance the hydrogen storage performance of MXenes, various strategies have been explored, such as the tuning of surface terminations, the intercalation of metal ions, and the creation of composite structures [73,74]. Wang et al. [75] prepared PrF_3_/Ti_3_C_2_ composite materials via a hydrothermal method. These materials exhibited excellent catalytic activity for MgH_2_ hydrogen storage. Adding 5 wt.% PrF_3_/Ti_3_C_2_ reduced the desorption onset temperature to 180 °C, a decrease of 107 °C compared to pristine MgH_2_. For MgH_2_-5 wt.% PrF_3_/Ti_3_C_2_, 7.0 wt.% hydrogen was rapidly desorbed within 3 min at 260 °C, and 6.6 wt.% hydrogen was rapidly absorbed within 36 s at 200 °C.

Layered double hydroxides (LDHs) are another class of 2D materials that have gained increasing attention for hydrogen storage applications [76]. LDHs consist of positively charged metal hydroxide layers intercalated with charge-balancing anions and water molecules. The general formula of LDHs is [M_1−x2_ + M_x3_ + (OH)_2_]_x_ + [An^−^]_x/n_·mH_2_O, where M^2+^ and M^3+^ are divalent and trivalent metal cations, respectively, and An^−^ is the interlayer anion. The tunable composition, high surface area, and ion-exchange properties of LDHs make them promising candidates for hydrogen storage. Table 4 summarizes the reservoir hydrogen performance indexes of LDH-based materials.

**Figure 2:**
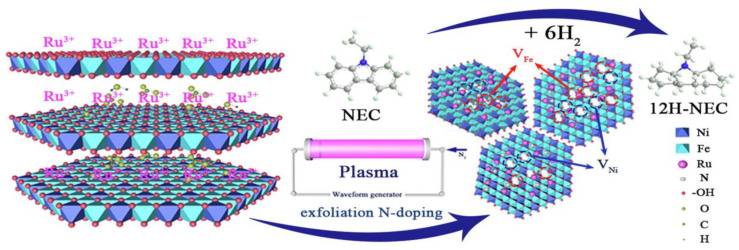
Mechanism diagram of glow discharge plasma preparation of Ru/Ni-Fe LDH [77].

Several studies have reported the hydrogen storage properties of LDHs, particularly those based on Mg and Al (MgAl-LDHs). To further enhance the hydrogen storage performance of LDHs, various strategies have been investigated, such as the optimization of the metal composition, the intercalation of functional anions, and the creation of hybrid structures [76]. Shi et al. [77] prepared ultrafine Ru nanoparticles (RuNPs) supported on nitrogen-doped exfoliated Ni-Fe layered double hydroxide nanosheets (Ru/Ni-Fe LDH) using nitrogen glow discharge plasma technology. Characterizations using FT-IR, XPS, XRD, AFM, TEM, SEM, and N_2_ physical adsorption-desorption analyses revealed that glow discharge plasma not only reduced Ru^3+^ to Ru but also exfoliated Ni-Fe LDH into nanosheets, generating defects and doping N in the layered structure. A possible mechanism of preparing Ru/Ni-Fe LDH catalyst is shown in Figure 2. The resulting RuNPs, with an average size of 1.81 nm, were well dispersed on the n-doped exfoliated Ni-Fe LDH nanosheets. Catalytic hydrogenation experiments of n-ethylcarbazole (NEC) on Ru/Ni-Fe LDH showed a 100% NEC conversion rate, a 97.43% 12H-NEC selectivity, a hydrogen storage capacity of 5.74 wt.%, and an apparent activation energy of 44.86 kJ/mol at 120 °C and 6 MPa in 80 min. 

In summary, MXenes and LDHs represent promising emerging nanomaterial platforms for hydrogen storage applications due to their unique structures, high surface areas, and tunable chemical properties. However, further research is needed to fully understand their hydrogen storage mechanisms, optimize their performance, and address the challenges associated with their synthesis, stability, and scalability.

## 4. Surface and Interface Engineering Strategies for Nanomaterials

### 4.1. Surface Modification for Enhanced Hydrogen Storage

Surface modification is an effective strategy to enhance the hydrogen storage properties of nanomaterials by tuning their surface chemistry, increasing the number of adsorption sites, and improving the hydrogen binding energy [78]. Various surface modification techniques have been explored, such as chemical functionalization, doping with heteroatoms, and the introduction of defects and vacancies.

Chemical functionalization involves the attachment of functional groups, such as amines, carboxyl groups, and metal nanoparticles, to the surface of nanomaterials. These functional groups can act as additional adsorption sites for hydrogen molecules and increase the binding energy through dipole-dipole interactions or orbital hybridization [79]. Mu et al. [80] modified carbon nanotubes (CNTs) via microwave plasma etching and Pd modification. The etching process increased defects on the nanotube walls, providing more hydrogen pathways in the interlayer and hollow interior of CNTs. At ambient temperature and a pressure of 10.728 MPa, etched CNTs exhibited higher hydrogen storage capacity than the original samples. Pd-modified CNTs showed a hydrogen storage capacity of 4.5 wt.%, approximately three times that of unmodified samples. Similarly, the functionalization of metal-organic frameworks (MOFs) with amine groups has been shown to increase their hydrogen storage capacity by creating additional adsorption sites and enhancing the binding energy through hydrogen bonding. Tsai et al. [81] investigated the hydrogen absorption behavior of CNTs modified by acidic etching and Pt particle deposition. Acidic etching increased surface defect density, opening the capped structure of CNTs and increasing active adsorption sites for physical adsorption of H_2_. Deposition of nanoscale Pt particles on CNTs using a conventional electrolyte facilitated chemical adsorption of hydrogen via the spillover mechanism. Supercritical CO_2_ (sc-CO_2_) treatment resulted in finer Pt particle size and more uniform distribution on the CNT surface, enhancing hydrogen storage capacity. CNTs modified with the combined process of acidic etching and sc-CO_2_ Pt deposition showed significantly enhanced hydrogen storage capacity to 2.7 wt.%.

Doping with heteroatoms, such as nitrogen, boron, and sulfur, is another effective approach to modify the surface properties of nanomaterials for hydrogen storage [82]. The incorporation of heteroatoms can create polarized sites on the surface, which enhance the hydrogen binding energy through electrostatic interactions. Graphene-based solid-state porous materials exhibit high specific surface area (2630 m^2^/g), porosity, light weight, high chemical and thermal stability (melting point 4510 K), and potential for economical and scalable production, showing promising applications for efficient hydrogen storage. While pristine graphene’s hydrogen storage performance is poor, doping with elements such as boron and nitrogen or modifying with transition metals can significantly improve its hydrogen storage properties. Additionally, graphene allows for surface curvature adjustment, facilitating reversible hydrogen storage systems with rapid kinetics [83]. The enhanced capacity was attributed to the increased binding energy between hydrogen molecules and the nitrogen-doped sites on the graphene surface. Similarly, the doping of boron into metal hydrides, such as LiBH_4_, has been shown to lower the desorption temperature and improve the reversibility by destabilizing the hydride phase.

The introduction of defects and vacancies on the surface of nanomaterials has also been explored as a means to enhance their hydrogen storage properties. Defects and vacancies can act as trapping sites for hydrogen molecules and increase the surface area and reactivity of the materials. Hydrogen can be inserted into the TiO_2_ nanotube wall layers to form a host-guest compound TiO_2−x_H_2_. When x ≤ 1.5, the hydrogen absorption temperature decreases as x increases. Additionally, the hydrogen adsorption rate increases with temperature. At 100 °C, the characteristic adsorption time for hydrogen in TiO_2_ nanotubes is several hours, with the formation rate of interlayer layers being limited by the diffusion of molecular hydrogen within the multilayer walls of TiO_2_ nanotubes [84]. Similarly, the introduction of nanoscale defects in boron nitride nanotubes (BNNTs) by ball milling has been shown to enhance their hydrogen storage capacity and binding energy by creating additional adsorption sites and increasing the polarity of the surface.

### 4.2. Nanocomposite Design for Synergistic Enhancement

The design of nanocomposites, which combine two or more materials with complementary properties, is another effective strategy to enhance the hydrogen storage performance of nanomaterials [85]. Nanocomposites can exhibit synergistic effects that are not achievable by the individual components, such as increased surface area, improved thermal and mechanical stability, and enhanced hydrogen adsorption/desorption kinetics.

**Figure 3:**
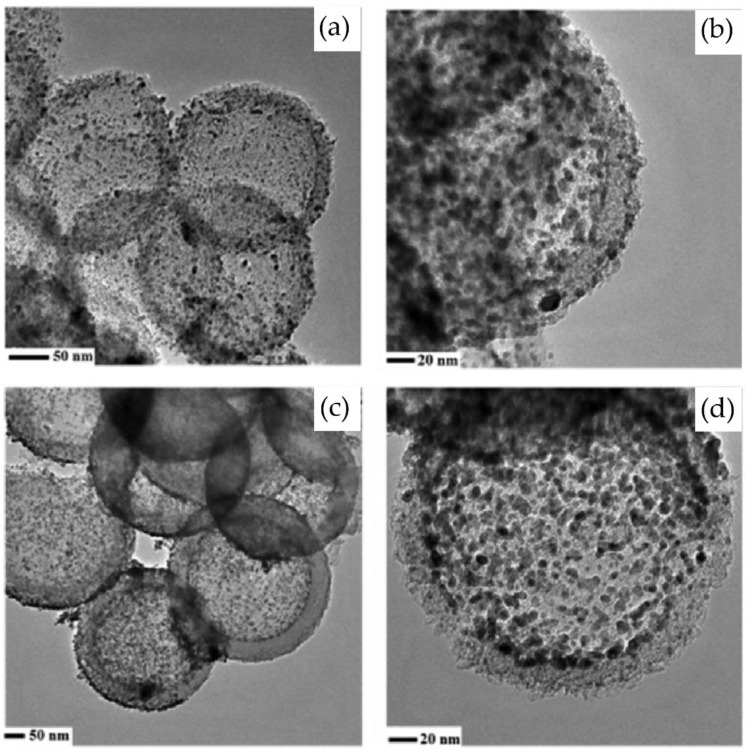
3-Pd@C_24 sample TEM images after the 1st (**a**,**b**) and 4th (**c**,**d**) adsorption/desorption cycles [87].

One common approach to design nanocomposites for hydrogen storage is the incorporation of metal nanoparticles into porous supports, such as carbon-based materials, MOFs, and COFs [86]. The metal nanoparticles can act as catalytic sites for hydrogen dissociation and spillover, while the porous supports provide high surface area and storage capacity. Baca et al. [87] modified disordered mesoporous hollow carbon spheres with Pd nanoparticles. At 40 °C and 45 bar, the Pd-loaded carbon spheres exhibited stronger H_2_ absorption capacity than the original materials. Despite a reduction in surface area and porosity after Pd addition, the H_2_ absorption increased with increasing Pd content and particle size. The sample morphology of the hydrogen adsorption of the second and fourth adsorption/desorption is shown in Figure 3, and the size of the two samples was 7.3 nm when the adsorption/desorption was studied.

Another approach to design nanocomposites for hydrogen storage is the integration of different types of nanomaterials with complementary properties, such as metal hydrides and porous materials. Surrey et al. [88] prepared micro/mesoporous carbon scaffolds using a salt-templating method and loaded them with 40 wt.% LiBH_4_ through melt infiltration. Thanks to nanoscale confinement, LiBH_4_ began to desorb hydrogen at 200 °C and primarily released hydrogen at 310 °C. Partial rehydrogenation was conducted under moderate conditions (100 bar and 300 °C).

The design of core-shell nanostructures is also an effective strategy to enhance the hydrogen storage properties of nanomaterials. Core-shell nanostructures consist of a core material encapsulated by a shell material with different chemical or physical properties. The shell material can protect the core from oxidation, prevent its aggregation, and modify its surface properties for enhanced hydrogen adsorption. Lu et al. [89] synthesized core-shell Mg@NaBH_4_ composites using arc plasma and vacuum vapor deposition techniques. The hydrogen activation energy for Mg in the composites was reduced to E_a_ = 60.1 kJ/mol H_2_, and the initial hydrogen desorption temperature of 245 °C was significantly lower than that of pure MgH_2_. This improvement was attributed to the core-shell structure, which facilitated more effective contact between the catalyst and Mg, providing additional nucleation sites for hydrogen adsorption. Additionally, the formation of MgB_2_ and substitution by Na atoms likely supported more grain boundaries, altering the electronic structure of Mg/MgH_2_ and significantly enhancing Mg’s hydrogen absorption properties.

### 4.3. Interface Engineering in Nanomaterials

Interface engineering is another important strategy to enhance the hydrogen storage properties of nanomaterials by tuning the interfacial structures and properties [90]. Interfaces play a crucial role in the hydrogen storage performance of nanomaterials, as they can act as active sites for hydrogen adsorption, dissociation, and diffusion [91]. The design and control of interfaces in nanomaterials can lead to enhanced hydrogen storage capacity, improved kinetics, and better cycling stability.

**Figure 4:**
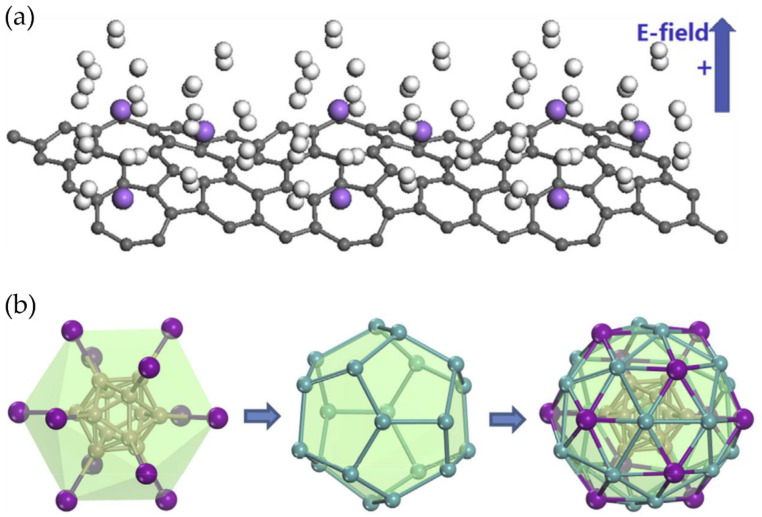
(**a**) The 3 × 1 supercell of the graphene-(Li_5_H_2_)_3_ structure [94]; (**b**) The construction of the core-cell structure of B_12_@Mg_20_Al_12_. Here, the icosahedral B_12_ structure (pink) is selected as the core structure, then each B atom connects with an Al atom (purple), forming a stable core-shell framework. The Mg atoms (cyan) are selected to decorate the B_12_Al_12_ framework, forming the core-shell structure of B_12_@Mg_20_Al_12_ [95].

One approach to engineer the interfaces in nanomaterials is the creation of heterojunctions between different materials with suitable band structures and catalytic properties [92]. Heterojunctions can facilitate the charge transfer and separation at the interface, leading to enhanced hydrogen adsorption and dissociation. Jiang et al. [93] designed an NL_4_Li-modified boron-doped graphene/hydrogenated magnesium heterojunction (NLi_4_BGra/MgH_2_) using density functional theory (Figure 4a). Molecular dynamics (MD) confirmed that this material could release H_2_ adsorbed by NLi_4_ at 298 K and hydrogen stored in MgH_2_ at 570 K. Subsequently, DFT combined with machine learning (ML) predicted 132 different magnesium alloy hydrides (Mg_1−x_MexH_2_) with various hydrogen storage capacities and desorption temperatures. The strategy of replacing MgH_2_ in NLi_4_-BGra/Mg_0.875_Me_0.125_H_2_ (Me = Li, Be) (Td = 400, 500 K) with Mg_1−x_Me_x_H_2_ was validated by verifying the root mean square deviation, diffusion coefficient, and hydrogen desorption trajectory, effectively adjusting the hydrogen desorption temperature range of the NLi_4_-BGra/MgH_2_-based heterojunction.

Another approach to engineer the interfaces in nanomaterials is the introduction of interfacial defects, such as grain boundaries, dislocations, and vacancies. Interfacial defects can act as trapping sites for hydrogen molecules and enhance the hydrogen binding energy by creating localized states and modifying the electronic structure of the materials. Zhang et al. [94] investigated the enhancement of hydrogen storage capacity by an electric field (e-field) perpendicular to a graphene surface with Stone-Wales (SW) defects and Li atom decoration using density functional theory calculations. Under an upward e-field of 0.01 au, each lithium ion on the SW defect graphene surface adsorbed a maximum of five H_2_ molecules, one more than without an electric field. SW defect graphene adsorbed hydrogen molecules primarily through polarization interactions. The calculated desorption temperature and molecular dynamics simulation results indicated that lithium-decorated graphene with SW defects is suitable for hydrogen storage applications under near-ambient conditions. Each Li atom can adsorb five H_2_ molecules, and the upward e-field stabilizes the dispersion of individual Li atoms, making this structure a better building block for polymers.

The design of core-shell nanostructures with engineered interfaces is also an effective strategy to enhance the hydrogen storage properties of nanomaterials [95]. By carefully selecting the core and shell materials and controlling the interfacial structures, it is possible to achieve enhanced hydrogen storage capacity, improved kinetics, and better cycling stability. Guo et al. [95] proposed a stable B_12_-containing icosahedral core-shell structure, B_12_@Mg_20_Al_12_, based on first-principles density functional theory (Figure 4b). Calculations showed that B_12_@Mg_20_Al_12_ could adsorb up to 146 hydrogen molecules, exhibiting a double-shell distribution and a hydrogen capacity of up to 23.7 wt.%, making it a promising material for high-capacity hydrogen storage.

In addition to the abovementioned strategies, the engineering of interfacial energy and strain has emerged as a promising approach to tuning the hydrogen storage properties of nanomaterials. Reddy et al. [96] prepared Pd/Ti/Mg films using a combination of resistance evaporation and electron beam evaporation. This composite material could release approximately 7 wt.% of hydrogen upon complete dehydrogenation at ~473 K. Tests indicated that the composite, after pulverization, could reversibly store hydrogen up to 2.2 wt.% over 18 cycles in the temperature range of 323–473 K. Edalati et al. [97] synthesized a nanostructured TiV alloy with a supersaturated bcc structure and ultra-high edge dislocation density using high-pressure torsion (HPT) from Ti and V powders. The numerous grain boundaries and dislocations serve as effective pathways for hydrogen diffusion, significantly accelerating the alloy’s kinetics and enabling the TiV alloy to absorb approximately 4 wt.% hydrogen at room temperature. Hongo et al. [98] produced Mg_2_Ni intermetallic compounds with various microstructures through different processing methods. The study demonstrated that increased grain boundaries and stacking faults enhanced the kinetics of Mg_2_Ni intermetallic compounds, allowing them to absorb 3.3 wt.% hydrogen at 150 °C, further validating the significant regulatory effects of interface energy and strain on the hydrogen storage performance of nanomaterials.

In summary, interface engineering is a powerful strategy to enhance the hydrogen storage properties of nanomaterials by tuning the interfacial structures and properties. The creation of heterojunctions, the introduction of interfacial defects, and the design of core-shell nanostructures with engineered interfaces are effective approaches to achieve enhanced hydrogen storage capacity, improved kinetics, and better cycling stability. However, further research is needed to fully understand the interfacial mechanisms and to optimize the interfacial structures for practical hydrogen storage applications.

## 5. Catalytic Enhancement by Nanocatalysts

### 5.1. Metal and Metal Oxide Nanocatalysts

Metal and metal oxide nanoparticles have been widely investigated as catalysts to enhance the hydrogen storage properties of nanomaterials. These nanocatalysts can facilitate the dissociation of hydrogen molecules, lower the activation energy for hydrogen adsorption/desorption, and improve the kinetics and reversibility of hydrogen storage.

Noble metal nanoparticles, such as Pt, Pd, and Ru, are among the most effective catalysts for hydrogen storage due to their high catalytic activity and selectivity. These nanoparticles can dissociate hydrogen molecules into atomic hydrogen, which can then spill over onto the surface of the support material and enhance the hydrogen storage capacity [99]. Campesi et al. [100] obtained C/Pd composites through chemical reduction. At 77 K and 1.6 MPa, the introduction of Pd did not increase hydrogen storage capacity, as hydrogen absorption was attributed to the physical adsorption of carbon. However, under room temperature and moderate pressure (0.5 MPa) conditions, carbon materials filled with 10 wt.% nanocrystalline Pd had eight times the hydrogen capacity of Pd-free materials.

**Figure 5:**
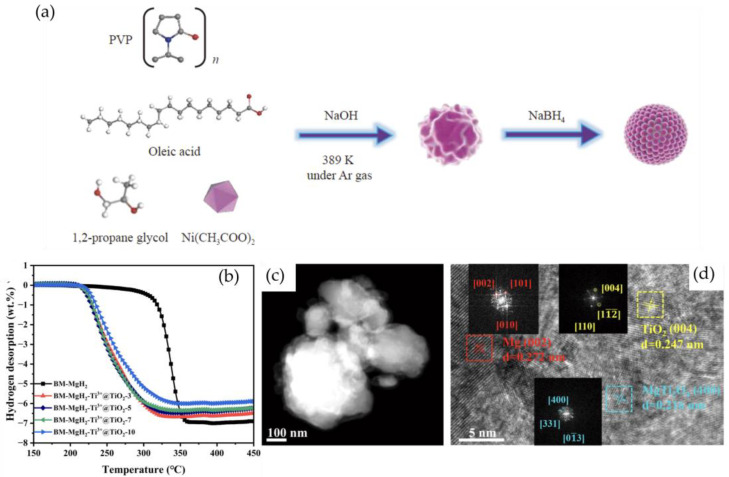
(**a**) Illustration of the synthesis of the nano-Ni composite [103]; (**b**) temperature dehydrogenation curve of ball milling composites; dehydrogenated BM-MgH_2_-Ti^3+^@TiO_2_-5 (**c**) TEM, (**d**) HRTEM [105].

Transition metal nanoparticles, such as Ni, Co, and Fe, are also effective catalysts for hydrogen storage due to their low cost and high abundance compared to noble metals [101]. These nanoparticles can catalyze the dissociation of hydrogen molecules and enhance the hydrogen adsorption/desorption kinetics [102]. Xu et al. [103] prepared highly dispersed Ni nanoparticles with an average particle size of 2.14 nm using a polyol reduction method (Figure 5a). The composite material, MgH_2_-10 wt.% nanonickel, began releasing H_2_ at 497 K and released approximately 6.2 wt.% H_2_ at 583 K. At 482 K and 3 MPa hydrogen pressure, the hydrogen absorption reached 5.3 wt.% within 1000 s. Additionally, the dehydrogenation and rehydrogenation activation energies of MgH_2_-10 wt.% nanonickel were reduced to (88 ± 2) and (87 ± 1) kJ·mol^−1^, respectively, significantly lower than those of pristine MgH_2_, improving the thermal stability of the MgH_2_-nanonickel system by 5.5 kJ/mol H_2_.

Metal oxide nanoparticles, such as TiO_2_, ZrO_2_, and CeO_2_, have also been investigated as catalysts for hydrogen storage due to their high surface area, good stability, and unique redox properties [104]. These nanoparticles can enhance the hydrogen storage capacity by providing additional adsorption sites and facilitating the dissociation of hydrogen molecules through the formation of oxygen vacancies. Yin et al. [105] synthesized Ti^3+^-self-doped 3D TiO_2_ hollow nanoboxes (Ti^3+^@TiO_2_) via a simple hydrothermal reaction. The initial hydrogen desorption temperature of the BM-MgH_2_-Ti^3+^@TiO_2_-5 composite was reduced to 205.4 °C, 49.2 °C lower than that of pristine MgH_2_ (Figure 5b). At 325 °C, the composite released 6.1 wt.% hydrogen within 20 min, and the desorption activation energy (E_a_) was significantly reduced to 57.2 ± 1.4 kJ/mol, showing excellent hydrogen desorption kinetics. Furthermore, the composite exhibited remarkable cycling stability, retaining 93.7% hydrogen capacity after 10 dehydrogenation/rehydrogenation cycles at 325 °C. The Ti^3+^@TiO_2_ composite exhibited excellent catalytic performance for hydrogen storage in MgH_2_, attributed to the rich oxygen vacancies and in situ formation of multivalent Ti species (Figure 5c,d).

### 5.2. Non-Metal Nanocatalysts and Their Catalytic Mechanisms

Non-metal nanocatalysts, such as boron nitride (BN), graphitic carbon nitride (g-C_3_N_4_), and black phosphorus (BP), have recently emerged as promising alternatives to metal-based catalysts for hydrogen storage [106]. These nanocatalysts possess unique electronic structures, high surface areas, and good chemical stability, which make them attractive for hydrogen storage applications [107].

**Figure 6:**
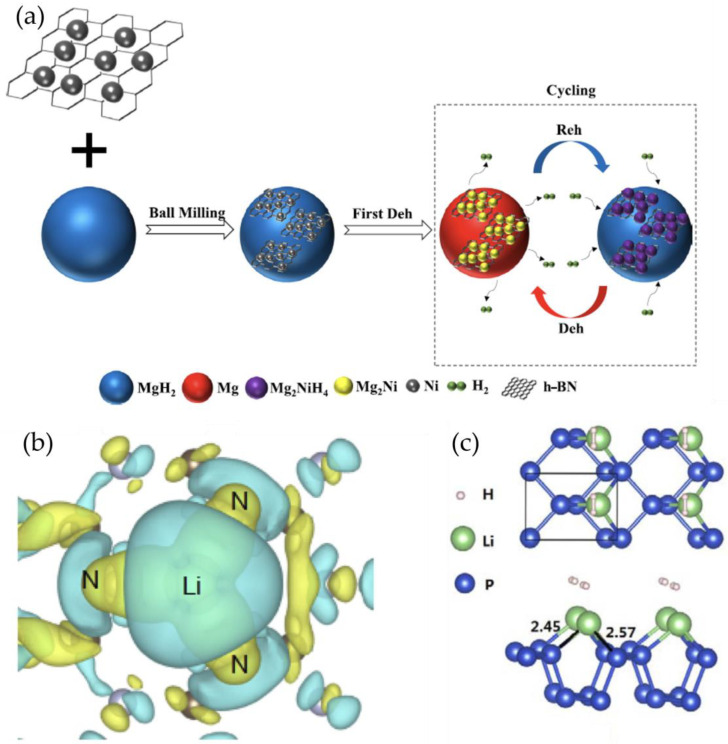
(**a**) A schematic graph of the synergistic effect of Ni_70_@BN during the dehydrogenation and rehydrogenation of MgH_2_ [30]. (**b**) Calculated charge density differences of the Li decorated g-C_3_N_4_ system; the turquoise color and yellow color represent charge accumulation and depletion (the value of isosurface is 0.0013 e/Å_3_) [112]. (**c**) Optimized geometric structures with adsorbed H_2_ in Li-decorated MBP. The 1 × 1 cell is outlined in the top view. Top and side view of Li-decorated MBP with a one H_2_ adsorption [113].

Boron nitride (BN) nanomaterials, such as BN nanosheets and BN nanotubes, have been investigated as catalysts for hydrogen storage due to their polar surface, which can facilitate the adsorption and dissociation of hydrogen molecules [108,109]. The presence of B-N bonds in BN nanomaterials creates a polarized surface with positively charged B atoms and negatively charged N atoms, which can attract hydrogen molecules through dipole-dipole interactions [110]. Jia et al. [30] prepared layered boron nitride nanosheets loaded with fine nickel particles using a solution chemical reduction method and introduced them into MgH_2_. As shown in Figure 6a, the Ni particles distributed on the BN surface provided numerous active sites, significantly improving the hydrogen storage performance of MgH_2_. The MgH_2_/Ni_70_@BN composite could quickly absorb 5.34 wt.% H_2_ within 25 s at 125 °C and release 6.21 wt.% H_2_ within 15 min at 300 °C. The dehydrogenation activation energy was reduced to 59.77 ± 3.96 kJ/mol, significantly lower than the 145.08 kJ/mol of pure MgH_2_. The anti-agglomeration effect of BN allowed the Ni_70_@BN-modified MgH_2_ to maintain excellent cycling performance even after 30 cycles. The synergistic effect between BN and Mg_2_Ni(H_4_) promoted the “hydrogen pump” effect, accelerating the adsorption/desorption kinetics of Mg/MgH_2_.

Graphitic carbon nitride (g-C_3_N_4_) is another promising non-metal catalyst for hydrogen storage due to its unique electronic structure, high nitrogen content, and good thermal stability [111]. The presence of nitrogen atoms in the g-C_3_N_4_ structure creates a delocalized π-conjugated system with a narrow bandgap, which can facilitate the charge transfer and enhance the catalytic activity for hydrogen storage [111]. Wei et al. [112] explored lithium-modified g-C_3_N_4_ based on first-principles calculations. As shown in Figure 6b, the adsorption energy of Li atoms on g-C_3_N_4_ was much greater than the cohesive energy of bulk Li, with each H_2_ molecule having a binding energy of approximately 0.29 eV, resulting in a predicted hydrogen storage capacity of about 9.2%, exceeding the U.S. Department of Energy’s target.

Black phosphorus (BP) nanomaterials, such as BP nanosheets and BP quantum dots, have recently emerged as promising catalysts for hydrogen storage due to their unique electronic structure, high surface area, and good chemical stability [113]. The presence of a bandgap in the BP structure creates a semiconducting property, which can facilitate the charge transfer and enhance the catalytic activity for hydrogen storage [114]. Li et al. [115] predicted that lithium-modified monolayer black phosphorus (MBP) could be a highly potential hydrogen storage medium based on systematic first-principles density functional calculations (Figure 6c). They found that pure MBP had weak H_2_ adsorption energy. Surprisingly, after lithium modification, its hydrogen storage performance was significantly enhanced.

In summary, metal and non-metal nanocatalysts play a crucial role in enhancing the hydrogen storage properties of nanomaterials by facilitating the dissociation of hydrogen molecules, lowering the activation energy for hydrogen adsorption/desorption, and improving the kinetics and reversibility of hydrogen storage. The catalytic mechanisms of these nanocatalysts involve the spillover effect, the formation of oxygen vacancies, the polarization of surface bonds, and the modulation of electronic structures. Further research is needed to optimize the catalytic performance of these nanocatalysts and to develop new catalytic systems for practical hydrogen storage applications.

## 6. Summary and Outlook

Solid-state hydrogen storage technology boasts significant advantages in high storage density and safety, yet it faces multiple barriers in scalability and industrial deployment. These barriers include high material costs, the energy demand for hydrogen release, the complexity of system design, safety management, and economic feasibility. The high cost and scarcity of materials limit large-scale applications, while improving hydrogen release and recovery efficiency requires more energy and complex thermal management systems. The complexity of system integration and design, along with volume and weight issues, also increases manufacturing and operational costs. Long-term stability and thermal management needs pose safety challenges, and achieving economic viability is crucial for market acceptance. Addressing these issues necessitates comprehensive efforts in material science, system engineering, and business models to promote the widespread application and industrialization of solid-state hydrogen storage technology.

### 6.1. Key Progress in Nanomaterials for Hydrogen Storage

In this review, we have provided a comprehensive overview of the recent advances in nanomaterials for hydrogen storage. We have discussed the fundamental principles and mechanisms of hydrogen storage in nanomaterials, highlighting the effects of size, surface area, and porosity on the hydrogen storage properties. We have also reviewed the typical nanomaterial systems for hydrogen storage, including lightweight metal nanoparticles, porous nanomaterials (carbon-based materials, MOFs/COFs, BNHs), and other emerging nanomaterial platforms (MXenes, LDHs).

Significant progress has been made in the development of nanomaterials for hydrogen storage in recent years. Nanostructuring of lightweight metals, such as Mg, Al, and Li, has led to enhanced hydrogen storage capacities and improved kinetics compared to their bulk counterparts. The use of porous nanomaterials, such as activated carbons, MOFs, COFs, and BNHs, has enabled high surface area and tunable porosity for efficient hydrogen adsorption. The emerging nanomaterial platforms, such as MXenes and LDHs, have shown promising hydrogen storage properties due to their unique 2D structures and surface functionalities.

Surface and interface engineering strategies, such as surface modification, nanocomposite design, and interface engineering, have been extensively investigated to enhance the hydrogen storage properties of nanomaterials. Surface modification techniques, such as chemical functionalization, heteroatom doping, and defect engineering, have been employed to increase the hydrogen binding energy and adsorption capacity. Nanocomposite design, such as the incorporation of metal nanoparticles into porous supports and the integration of different nanomaterials, has led to synergistic enhancement in hydrogen storage performance. Interface engineering, such as the creation of heterojunctions and the introduction of interfacial defects, has been explored to tune the interfacial structures and properties for enhanced hydrogen storage.

Catalytic enhancement by metal and non-metal nanocatalysts has also been widely investigated to improve the hydrogen storage properties of nanomaterials. Noble metal nanoparticles (Pt, Pd, Ru), transition metal nanoparticles (Ni, Co, Fe), and metal oxide nanoparticles (TiO_2_, ZrO_2_, CeO_2_) have been employed as effective catalysts to facilitate the dissociation of hydrogen molecules and enhance the hydrogen adsorption/desorption kinetics. Non-metal nanocatalysts, such as BN, g-C_3_N_4_, and BP, have emerged as promising alternatives to metal-based catalysts due to their unique electronic structures and surface properties.

### 6.2. Challenges and Opportunities for Nanomaterial-Based Hydrogen Storage

Despite the significant progress made in the development of nanomaterials for hydrogen storage, several challenges still need to be addressed for their practical applications. One major challenge is the limited gravimetric and volumetric hydrogen storage capacities of current nanomaterials, which are still far from the ultimate targets set by the U.S. Department of Energy (7.5 wt.% and 70 g/L by 2025). The development of new nanomaterial systems with higher hydrogen storage capacities and the optimization of existing nanomaterials through surface and interface engineering are crucial to overcome this challenge.

Other challenges are the slow kinetics and poor reversibility of hydrogen adsorption/desorption in many nanomaterial systems, especially those based on chemisorption. The high activation energy and the strong binding of hydrogen to the nanomaterial surface often lead to slow hydrogen uptake and release rates and limited cyclability. The development of advanced catalytic systems and the engineering of surface and interface structures are important strategies to improve the kinetics and reversibility of hydrogen storage in nanomaterials.

The scalability and cost-effectiveness of nanomaterial synthesis and processing are also significant challenges for practical hydrogen storage applications. Many nanomaterial systems are currently synthesized by complex and expensive methods, such as chemical vapor deposition, arc discharge, and laser ablation, which limit their large-scale production and commercialization. The development of simple, scalable, and cost-effective synthesis methods, such as ball milling, wet chemical synthesis, and electrochemical deposition, is crucial for the practical implementation of nanomaterial-based hydrogen storage systems.

The stability and safety of nanomaterials under practical operating conditions, such as high pressure, high temperature, and long-term cycling, are also important challenges for hydrogen storage applications. Nanomaterials often suffer from structural and chemical instability, such as agglomeration, oxidation, and leaching, which can degrade their hydrogen storage performance and pose safety hazards. The development of robust and stable nanomaterial systems through surface protection, nanostructure optimization, and compositional tuning is essential for their practical applications.

Despite these challenges, there are also significant opportunities for nanomaterial-based hydrogen storage in the future. The increasing demand for clean and sustainable energy sources, such as hydrogen fuel cells, provides a strong driving force for the development of advanced hydrogen storage materials and technologies. The recent progress in nanomaterial synthesis, characterization, and computational modeling also offers new tools and insights for the rational design and optimization of nanomaterials for hydrogen storage.

### 6.3. Strategies and Directions for Material Design and Optimization

Based on the challenges and opportunities discussed above, several strategies and directions can be proposed for the future design and optimization of nanomaterials for hydrogen storage.

1. Developing new nanomaterial systems with high surface area, tunable porosity, and strong hydrogen binding sites. This can be achieved by exploring new compositions, structures, and functionalities of nanomaterials, such as novel MOFs, COFs, and 2D materials with tailored pore sizes and surface chemistries.
2. Optimizing the surface and interface structures of existing nanomaterials through advanced engineering strategies. This includes the fine-tuning of surface functionalities, the creation of heterojunctions and interfacial defects, and the design of core-shell and hierarchical nanostructures with synergistic effects.
3. Enhancing the catalytic activity and selectivity of nanocatalysts for hydrogen storage through rational design and engineering. This involves the development of new catalytic systems based on earth-abundant elements, the optimization of catalyst-support interactions, and the engineering of electronic and geometric structures of catalysts.
4. Developing simple, scalable, and cost-effective synthesis methods for nanomaterials with controlled size, shape, and composition. This can be achieved by exploring new synthesis routes, such as mechanochemical synthesis, microwave-assisted synthesis, and bio-inspired synthesis, and by optimizing the synthesis parameters and conditions.
5. Improving the stability and safety of nanomaterials under practical operating conditions through surface protection, nanostructure optimization, and compositional tuning. This includes the development of protective coatings, the engineering of stable nanostructures, and the incorporation of stabilizing additives and dopants.
6. Integrating computational modeling and machine learning techniques with experimental studies for the rational design and screening of nanomaterials for hydrogen storage. This involves the development of accurate and efficient computational models, the integration of data-driven approaches with physical insights, and the establishment of materials databases and platforms for knowledge sharing and collaboration.

In conclusion, nanomaterials have shown great promise for hydrogen storage applications due to their unique size-dependent properties, high surface area, and tunable porosity. Significant progress has been made in the development of various nanomaterial systems, such as lightweight metal nanoparticles, porous nanomaterials, and emerging 2D materials, for efficient hydrogen storage. Surface and interface engineering strategies, such as surface modification, nanocomposite design, and interface engineering, have been extensively investigated to enhance the hydrogen storage properties of nanomaterials. Catalytic enhancement by metal and non-metal nanocatalysts has also been widely explored to improve the kinetics and reversibility of hydrogen storage in nanomaterials.

Despite the progress made, several challenges still need to be addressed for the practical applications of nanomaterial-based hydrogen storage systems, such as the limited hydrogen storage capacities, slow kinetics, poor reversibility, high cost, and safety concerns. To overcome these challenges and realize the full potential of nanomaterials for hydrogen storage, further research and development efforts are needed in the rational design, synthesis, characterization, and optimization of nanomaterials with high performance, low cost, and good stability. The integration of computational modeling, machine learning, and experimental studies is also crucial for accelerating the discovery and development of new nanomaterials for hydrogen storage.

With the increasing demand for clean and sustainable energy sources and the rapid advances in nanomaterial science and technology, it is expected that nanomaterial-based hydrogen storage systems will play an important role in the future hydrogen economy. The continued research and development of nanomaterials for hydrogen storage will not only contribute to the realization of a sustainable and low-carbon future but also offer new opportunities for the advancement of materials science and engineering.

## Figures and Tables

**Table 1:** Comparison of hydrogen storage properties of lightweight metal-based materials.

Material	Theoretical Capacity (wt.%)	Desorption Temperature (°C)	Kinetics
MgH_2_	76	300–400	Slow
AlH_3_	10.1	150–200	Fast
LiAlH_4_	10.5	120–250	Fast
LiNH_2_	11.5	250–300	Slow
LiBH_4_	18.5	300–450	Slow
NaAlH_4_	7.4	180–200	Moderate

**Table 2:** Hydrogen storage properties of porous.

Material	Surface Areas (m^2^/g)	Pore Size (nm)	H_2_ Uptake (wt.%, 77 K/1 Bar)	H_2_ Uptake (wt.%, RT/100 Bar)
Activated carbon	3000	0.5–2.0	1.5–2.0	0.5–0.8
Carbon nanotubes	200–400	10–50	1.2–2.4	0.2–0.4
Graphene	500–1200	0.5–10	1.0–1.6	0.1–0.3
MOF-5	3800	0.8	5.2	1.3
MOF-177	4500	1.1	7.5	0.6
COF-102	3620	1.2	7.2	1.5
COF-103	4210	0.9	7.0	1.2
Porous BN	1900	1–10	2.6	-

**Table 3:** Hydrogen storage properties of vanadium-based MXene materials.

Material	Synthesis Method	Surface Area	H_2_ Uptake (wt.%, 77 K/1 Bar)	H_2_ Uptake (wt.%, RT/100 Bar)
V_2_C	HF etching of V_2_AlC	8	2.1	1.8
V_2_C(OH)_2_	Alkalization of V_2_C	15	3.5	2.6
V_2_C(ONa)_2_	Sodiation of V_2_C	23	4.2	3.3
Mo-doped V_2_C(OH)_2_	Hf etching + Mo doping	27	4.8	3.9
V_4_C_3_(OH)_2_	HF etching of V_4_AlC_3_	19	3.8	3.0

**Table 4:** Comparison of hydrogen storage properties of LDH-based materials.

Material	Synthesis Method	Surface Area (m^2^/g)	Pore Size (nm)	H_2_ Uptake (wt.% RT/60 Bar)
Mg_2_Al-LDH	Coprecipitation	45	0.5–1.0	0.6
Mg_2_Al-LDH/GO	In situ growth on GO	92	1.0–2.0	1.4
Mg_2_Al-LDH/CNT	In situ growth on CNT	116	1.5–3.0	2.0
Ni_2_Al-LDH	Coprecipitation	37	0.5–1.5	0.4
Ni_2_Al-LDH	Self-assembly	156	2.0–4.0	2.4
Co_2_Al-LDH	Coprecipitation	29	0.5–1.2	0.3
Co_2_Al-LDH/g-C_3_N_4_	Electrostatic assembly	103	1.2–2.5	1.8
